# BM-Map: an efficient software package for accurately allocating multireads of RNA-sequencing data

**DOI:** 10.1186/1471-2164-13-S8-S9

**Published:** 2012-12-17

**Authors:** Yuan Yuan, Clift Norris, Yanxun Xu, Kam-Wah Tsui, Yuan Ji, Han Liang

**Affiliations:** 1 Graduate Program in Structural & Computational Biology & Molecular Biophysics, Baylor College of Medicine, Houston, TX 77030, USA; 2 Department of Bioinformatics and Computational Biology, The University of Texas MD Anderson Cancer Center, Houston, TX 77030, USA; 3 Division of Quantitative Sciences, The University of Texas MD Anderson Cancer Center, Houston, TX 77030, USA; 4 Department of Statistics, Rice University, Houston, TX 77005, USA; 5 Department of Biostatistics, The University of Texas MD Anderson Cancer Center, Houston, TX 77030, USA; 6 Department of Statistics, University of Wisconsin - Madison, Madison, WI 53792, USA; 7 Current address, NorthShore University HealthSystem, Evanston, IL 60201, USA

## Abstract

**Background:**

RNA sequencing (RNA-seq) has become a major tool for biomedical research. A key step in analyzing RNA-seq data is to infer the origin of short reads in the source genome, and for this purpose, many read alignment/mapping software programs have been developed. Usually, the majority of mappable reads can be mapped to one unambiguous genomic location, and these reads are called unique reads. However, a considerable proportion of mappable reads can be aligned to more than one genomic location with the same or similar fidelities, and they are called "multireads". Allocating these multireads is challenging but critical for interpreting RNA-seq data. We recently developed a Bayesian stochastic model that allocates multireads more accurately than alternative methods (Ji *et al. *Biometrics 2011).

**Results:**

In order to serve a greater biological community, we have implemented this method in a stand-alone, efficient, and user-friendly software package, BM-Map. BM-Map takes SAM (Sequence Alignment/Map), the most popular read alignment format, as the standard input; then based on the Bayesian model, it calculates mapping probabilities of multireads for competing genomic loci; and BM-Map generates the output by adding mapping probabilities to the original SAM file so that users can easily perform downstream analyses. The program is available in three common operating systems, Linux, Mac and PC. Moreover, we have built a dedicated website, http://bioinformatics.mdanderson.org/main/BM-Map, which includes free downloads, detailed tutorials and illustration examples.

**Conclusions:**

We have developed a stand-alone, efficient, and user-friendly software package for accurately allocating multireads, which is an important addition to our previous methodology paper. We believe that this bioinformatics tool will greatly help RNA-seq and related applications reach their full potential in life science research.

## Background

In recent years RNA-seq has become a popular and powerful approach for transcriptome profiling [1,2]. Using this approach, millions of short reads are generated from RNA samples by next-generation platforms such as Illumina Solexa and ABI SOLiD. Due to such sequence-based "digital output", RNA-seq not only allows a more accurate quantification of gene expression than conventional microarrays [2-4], but also is able to characterize other aspects of transcriptome such as alternative splicing [5,6], gene fusion [7], RNA editing [8] and expressed alleles [9,10].

A key step in the analysis of RNA-seq data is read mapping, the goal of which is to infer the origin of short reads in the source genome. For this purpose, many software programs have been developed such as Bowtie [11], BFAST [12] and MAQ [13]. These programs align each read independently to a reference genome based on sequence similarity. As a result, the majority of mappable reads (e.g., 70~80%) are mapped to one unambiguous genomic location, and these reads are called "unique reads". On the other hand, a considerable proportion of mappable reads can be aligned to more than one genomic location with the same or similar fidelities, and they are called "multireads". Currently, a common practice in the community is to use unique reads only for downstream analysis. This practice not only discards potentially useful information, but also introduces an underestimation bias for quantifying expression of genes with highly redundant sequences (e.g., young duplicated genes).

Therefore, we consider allocating multireads is a critical issue in the RNA-seq analysis, which has received relatively little attention in literature. One pioneering and influential method is "the proportional method" developed by Mortazavi et al. (2008) [14] in which unique reads are first mapped, and then multireads are allocated to competing locations in proportion to the numbers of mapped unique reads associated with the locations. The key idea of the proportional method is to borrow the information from unique reads in the dataset to guide the allocation of multireads. Motivated by such an idea, we recently proposed a Bayesian stochastic model for allocating multireads, which takes full advantage of information stored in the mapped unique reads [15]. The simulation results show that our method has better performance than other allocating methods [15]. In order to serve a greater biological community, we have implemented this method in a stand-alone, efficient and user-friendly software package, "BM-Map", which may help RNA-seq and related applications reach their full potential in life science research.

### Implementation

#### Overview of the BM-Map algorithm

**Figure 1:**
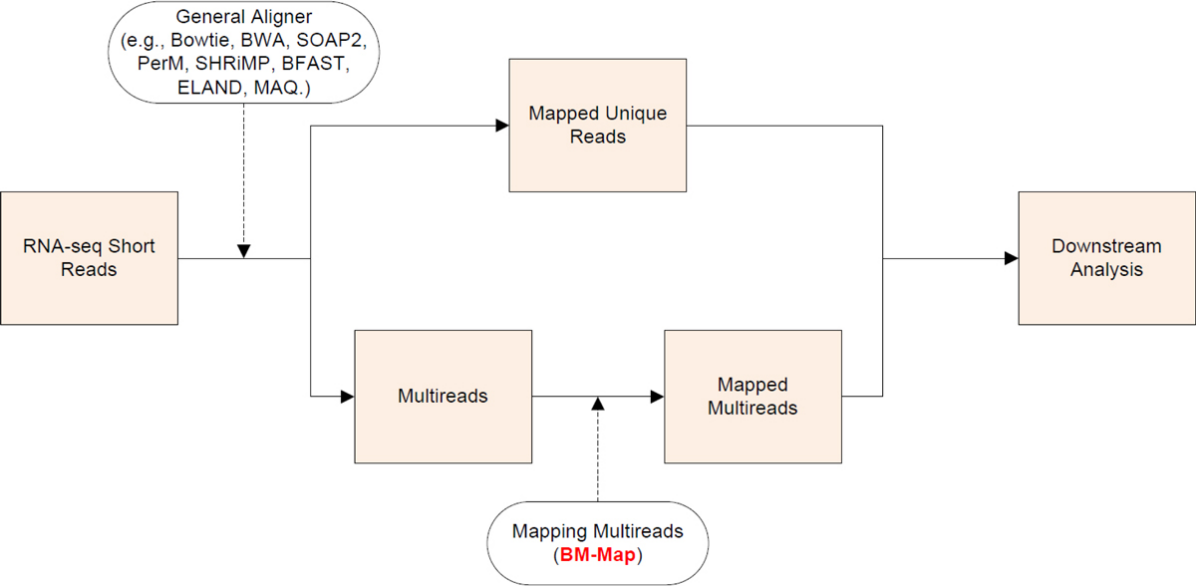
**The analytic pipeline of RNA-seq data with an additional refinement step on allocating multireads**.

Figure 1 shows where our BM-Map program stands in the pipeline of RNA-seq data analysis. RNA-seq short reads are first mapped to the reference genome, and based on the sequence alignments, mappable reads are classified into unique reads and multireads (for pair-end reads, the read pairs can be classified into uniquely mapped pairs and those mapped to multiple loci). Then, our BM-Map program refines the mapping of multireads by computing their assignment probabilities to competing loci.

The details about the underlying probability model and algorithm are depicted in our previous study [15], and here we provide a quick review. Suppose a multiread can be aligned to several genomic locations with the same or similar mismatch numbers, and each competing location is associated with a set of unique reads (these unique reads usually partially overlap with a competing location). Given the observed data, our BM-Map method computes a "posterior" probability for mapping the multiread to each competing location. According to Bayes' rule, the posterior distribution can be computed in terms of two quantities: (i) the probability of observing the mismatching pattern of the multiread if it is generated from a specific genomic location, referred to as the "likelihood" of the data; and (ii) an expectation about the distribution of the multiread before observation of data, referred to as the "prior" distribution.

**Figure 2:**
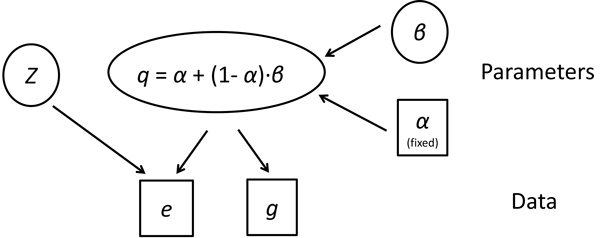
**The graphic model summarizing the parameters and data in our BM-Map algorithm**. In the model, *e *and *g *are the observed data, representing the mismatch patterns of multiread and related unique reads, respectively; α is sequencing error rate by considering all the unique reads in the dataset; β is the probability of mismatch due to hidden nucleotide variations; *q *is the probability of mismatch based on α and β; and *Z *is the prior distribution of reads mapping, which is set in proportion to the numbers of unique reads associated with competing loci.

In our model, the likelihood can be calculated based on the probability of mismatch at each nucleotide position of the multiread (*q*). We assume that the observed mismatches (*e *for multiread and *g *for unique reads) come from two resources. The first one is sequencing error (α), and we estimate quality-score specific error rate by considering all the unique reads in the dataset. The second source is called hidden nucleotide variations (β), which is mainly due to that RNA-seq reads are typically mapped to a public reference genome rather than the actual sample genome. Hence, variations between the two genome versions (e.g., SNPs) may cause some observed mismatches between the reads and the reference genome. Given the unique reads associated with a competing location, we model the uncertainty of hidden nucleotide variations using Markov chain Monte Carlo (MCMC) simulations. The prior distribution of reads mapping (*Z*) is set in proportion to the numbers of unique reads associated with competing loci, which essentially follows the main idea in Mortazavi et al. (2008) [14]. Figure 2 summarizes the relationships among parameters and data in our model.

#### Software implementation

Because the amount of RNA-seq data is large and our Bayesian method relies on extensive numerical computations for statistical inference, we implemented our method in a C++ programming written software package. To reduce the computer memory requirement for the calculations, we carefully examined the aligned data to minimize the size of the data structures that were held in memory simultaneously. To reduce lengthy execution times, we optimized the search algorithms used to process the reads. Finally, we achieved a further reduction in wall-clock runtime by running the calculations on multiple parallel threads of execution utilizing the capabilities typical of a modern desktop computer (i.e., Intel multi-core CPU). Currently, for an RNA-seq dataset with 8 million post-aligned short reads, our BM-Map program takes 3~4 hours to complete allocating the multireads in a single Windows 64-bit desktop machine (Intel Core i7@2.93 GHz, 16 GB memory), which is generally comparable to the computing time of mapping short reads by other methods.

**Figure 3:**
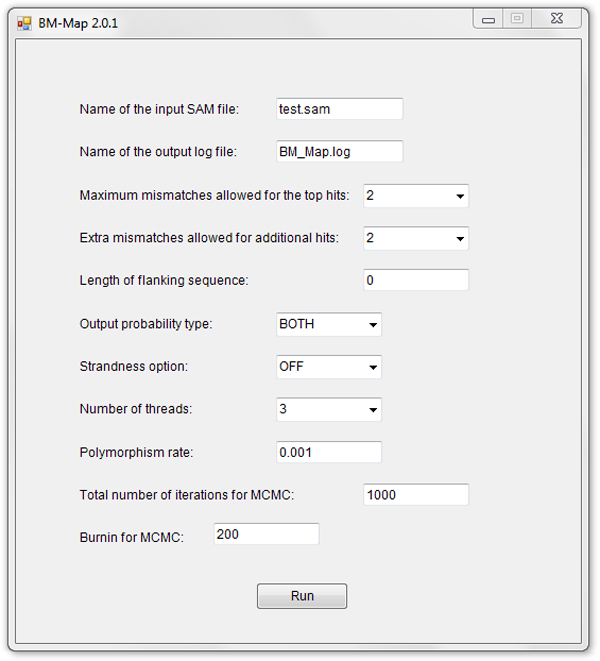
**The graphic user interface of BM-Map**.

We have made BM-Map a user-friendly software package. BM-Map takes SAM format (Sequence Alignment/Map) [16], the most popular read alignment format, as the standard input. Then based on the Bayesian model with parameters defined in a simple configuration file, BM-Map calculates mapping probabilities of multireads for competing genomic loci; and it generates the output in a SAM-like format by adding mapping probabilities to the original input file. The mapping probabilities can be easily parsed and used as weights in downstream analyses. Users can run BM-Map through a graphical user interface (as shown in Figure 3) or command lines (which is convenient for processing a large number of input files). We have provided the stand-alone executables for three common operating platforms (Linux, Mac and PC) at http://bioinformatics.mdanderson.org/main/BM-Map. The website also contains detailed tutorials and references about the BM-Map software.

## Results and discussion

Based on a Bayesian stochastic model we previously proposed, we have developed an efficient and user-friendly software package for allocating RNA-seq multireads, which takes full advantage of the information stored in the unique reads, including sequencing error profiles, the likelihood of hidden nucleotide variations (e.g., SNPs), and the expression level of competing locations. After reads are aligned by a general aligner, a mapping refinement step on multireads using our program would further improve the accuracy of gene expression quantification.

Obviously, the effect of allocating multireads on gene expression is related to next-generation sequencing techniques. With the increase of read length, whether to include multireads in expression quantification would become less important. Nevertheless, through our analysis on RNA-seq datasets with different read lengths (from 36~76 nt), the effect of multireads on certain genes (e.g., young duplicated genes) appear to be non-negligible given current sequencing technologies (additional file 1). Moreover, among the leading sequencing platforms, the read length of ABI SOLiD has been stable at 50 nt for some time. Finally, there are huge amounts of earlier generated RNA-seq reads in the public domain, and our program may be a key tool for data mining on such data.

The value of allocating multireads using BM-Map also depends on the organisms and genes of interest. Our program would have a relatively large effect for (i) organisms underwent several rounds of whole-genome duplications such as plants [17], (ii) genes with closely related paralogs or with recent pseudo-genes and (iii) transposon-derived transcripts [18]. Moreover, since our BM-Map considers the uncertainty due to hidden nucleotide variations, it could be more useful for the species with a high polymorphism rate. This is particularly true when the original genome under survey is not available and that of a closely related species is used as surrogacy for read mapping. In addition, our BM-Map will be highly valuable for other RNA-seq related applications, such as RNA immunoprecipitation sequencing (RIP-seq), cross-linking immunoprecipitation sequencing (CLIP-seq) [19] and 3'-end sequencing (3-seq) [20]. In future, we will further speed up our BM-Map algorithm and make the software more convenient for processing pair-end reads.

## Conclusions

We have developed a stand-alone and user-friendly software package, BM-Map, which can accurately allocating a large amount of RNA-seq multireads in an efficient way. We expect that this useful bioinformatics tool would help RNA-seq and its related applications reach their full potential in life sciences and biomedical research.

## Availability and requirements

**• Project name: **BM-Map

**• Project home page: **http://bioinformatics.mdanderson.org/main/BM-Map

**• Operating system(s): **Linux, Mac and PC

**• Programming language: **C++

**• Other requirements: **No

**• License: **No

**• Any restrictions to use by non-academics: **No

## List of Abbreviations

CPU: central processing unit; MCMC: Markov Chain Monte Carlo; nt: nucleotide; RNA-seq: RNA sequencing; RPKM: reads per kilobase of exon model per million mapped reads; SAM: sequence alignment map; SNP: single-nucleotide polymorphism; RIP-seq: RNA immunoprecipitation sequencing; CLIP-seq: cross-linking immunoprecipitation sequencing; 3-seq: 3'-end sequencing.

## Competing interests

The authors declare that they have no competing interests.

## Authors' contributions

Y.J. and H.L. designed the research and drafted the manuscript. Y.Y. and C.N. wrote the programming code for the software and built the website. Y.X. and K.-W.T. optimized the algorithm for efficiency. All authors read and approved the final manuscript.

## Supplementary Material

Additional file 1 **Effect of multireads on the expression quantification of different gene groups**. Click here for file
