# Supplementary material for: BM-Map: an efficient software package for accurately allocating multireads of RNA-sequencing data
Source: BMC Genomics. 2012 Dec 17;13(Suppl 8):S9. doi: 10.1186/1471-2164-13-S8-S9 (PMC3535707; doi:10.1186/1471-2164-13-S8-S9)

Additional File 1. Effect of multireads on the expression quantification of different groups of genes.

We obtained three RNA-seq datasets from Wang et al. (36 nt), Pickrell et al. (46 nt) and Wontan et al. (76 nt) and aligned them with Bowtie, resulting in mappable reads of 5.22 millions, 5.59 millions and 8.85 millions, respectively. Unique reads are defined as those only with one mapped location or the second best hit containing more than two extra mismatches. Normalized RPKM difference = $(RPKM_{BM\text{-}Map}-RPKM_{Unique})\times 2/(RPKM_{BM\text{-}Map}+RPKM_{Unique})$. Sequence identity with the closet paralogs was obtained from Ensembl (version 50), and the value of singleton genes was set as 0.

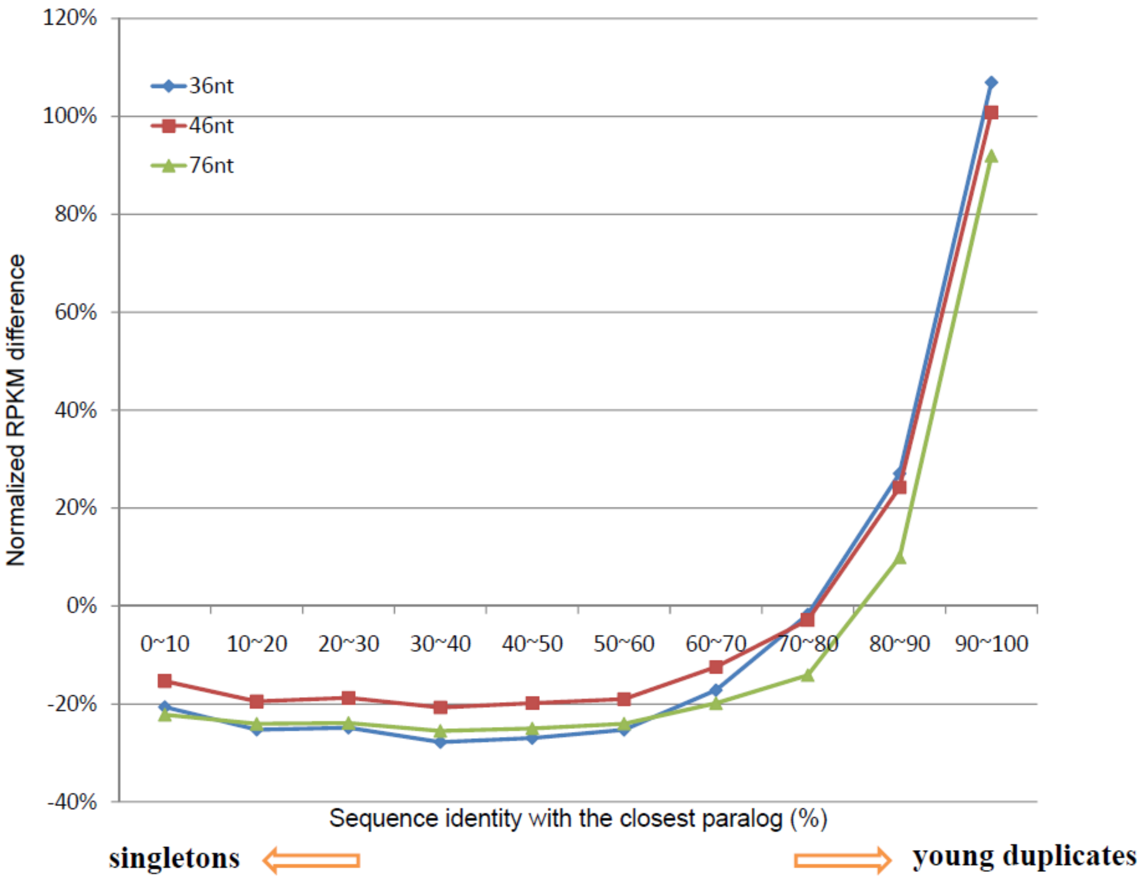

Supplement: Additional file 1 — Effect of multireads on the expression quantification of different gene groups. [file 1471-2164-13-S8-S9-S1.pdf]
